# Fantastic voyage: Catheter-based quantification of tracer distribution on a miniature scale

**DOI:** 10.1007/s12350-020-02379-8

**Published:** 2020-10-06

**Authors:** James T. Thackeray

**Affiliations:** Department of Nuclear Medicine, Hannover Medical School, Carl Neuberg Str 1, Hannover, 30625 Germany

The expansion of the nuclear cardiology armamentarium to encompass a wider range of tracers targeted to specific molecular processes offers opportunities for precise monitoring of disease progression and guidance of therapy.^1^ While some processes affect the global myocardium, many such as post-ischemic inflammation, infection, or fibroblast activation tend to be regional.^2^,^3^ Particularly subtle disease may also be below the detection limit of conventional imaging techniques. Moreover, localized pathology poses challenges to delivery of targeted cell, gene, or drug therapy, which may require precise targeting to evoke optimal benefit. As such, the limitations of resolution, regional heterogeneity, and mixed cellular substrates complicate interpretation of conventional radionuclide images, and raise the question whether other strategies could more precisely quantify regional tracer distribution.

In the current issue of the *Journal of Nuclear Cardiology*, Stendahl and colleagues describe a novel diagnostic device for minimally invasive tissue-level detection of tracer distribution in the myocardium, with a vision toward guided regional therapeutic intervention.^4^ The miniature plastic scintillator effectively discriminates β+ and β− activity over γ-radiation, which could precisely pinpoint the spatial binding of a positron-emitting tracer. In a pig heart after balloon-induced myocardial infarction, heterogeneous uptake ^18^F-fluorodeoxyglucose (^18^F-FDG) was observed by ex vivo PET imaging, thought to reflect regional inflammatory cell infiltration to the infarct and border zone territories. The regional disparity in signal was confirmed by spatial mapping using the β-detection catheter, providing a matrix distribution of activity content. Segment-to-segment activity established reasonable correlations between ex vivo PET, gamma well counting, and catheter-based measurements supporting the accuracy of the latter.

The strength of the manuscript lies in the ingenuity of the approach, which tackles the challenge of regional tracer uptake heterogeneity using a minimally invasive local measurement. While the analysis is limited to only two animals and selected areas in these infarct hearts, validation by conventional (albeit ex vivo) PET and well counting indicates the capacity of the β-detector catheter to define spatial disparities of tracer accumulation. However, it is disappointing that this potential is not explored further, e.g., by confirmation of cellular substrate by histopathology. Indeed, while typical investigation requires alignment of adjacent tissue sections for ex vivo autoradiography, histology, and immunohistochemistry,^5^,^6^ the β-detection catheter could better define the target regions of tracer enrichment to characterize the signal. A growing range of targeted inflammation radiotracers have displayed divergent uptake patterns in specific leukocyte subtypes in vitro with variable uptake in other cardiac cell subtypes including myocytes and fibroblasts.^7^,^8^ But in many cases the exact target cell type for these radiotracers remains equivocal. The development of radiotracers targeting reparative macrophage subtypes via mannose receptor,^9^ which are difficult to isolate within the mixed leukocytes in the damaged region, may be better identified by combining spatial catheter-based measurements with histology. It remains unclear how the spatial mapping of activity aligns to leukocytes, myocytes, and other support cells, which would provide greater support for the accuracy and applicability of this approach. Accordingly, further study should combine regionally focused β-detection with histopathology to determine the feasibility of image fusion and advance understanding of tracer substrates.

This limitation in regional characterization is compounded by the reliance on ^18^F-FDG distribution for proof-of-concept. ^18^F-FDG is notoriously promiscuous, which hampers the interpretation of the meaning of the signal. Indeed, numerous studies have demonstrated the inefficacy of typical suppression methods, especially in actively remodeling or acute infarct myocardium, generating a mixed substrate for the ^18^F-FDG signal including inflammatory leukocytes and metabolically compromised cardiomyocytes,^3^,^10^ though the regional heterogeneity observed in the porcine hearts and fasting protocols suggest preferential accumulation in infiltrating macrophages. Nonetheless, a more specific radiotracer could give greater clarity to the accuracy of the measurements.

A theoretical benefit of the β-detection catheter is the possibility to selectively distinguish β+ decay from γ decay. Such detection might provide a more accurate localization of the signal by identifying the emitted particle rather than the annihilation event. With a rising prevalence of transition metal radiochemistry in cardiovascular molecular imaging,^8^ reconstruction accuracy is affected by wider positron range and partial volume effects. Regional mapping of activity concentration insulates the measurement from partial volume effects, particularly with thinning ventricle walls. In principle, the β-detection at the tissue level overcomes the spatial challenge of physics, accurately defining tracer distribution segment-by-segment.

Sensitivity poses a further challenge, wherein conventional PET imaging of inflammation after myocardial infarction has generally not attempted to discern between regionally robust or subtle inflammatory content. In conditions like myocardial infarction where the imaging target (i.e., leukocytes) is also elevated in the circulation, delineation of subtle tissue inflammation can be challenging, especially with transition metal isotopes. While the total inflammatory signal on PET imaging can predict functional outcome,^5^,^11^,^12^ subtle disease, and its role in the progression of heart failure is more difficult to assess. It is conceivable that β-detection catheter measurements could exhibit higher sensitivity for subtle disease, but this would require validation in vivo.

Indeed, it is problematic that the analysis is by necessity conducted entirely ex vivo. Clearly one benefit for the β-detection catheter is the possibility for minimally invasive measurement, providing a more accurate virtual biopsy on the miniature scale. It will be essential to demonstrate the equivalence of in vivo measurements, replete with cardiac motion to confirm the accuracy of catheter-based measurements in the real clinical situation. This will further allow characterization of β-detector sensitivity for activity in tissue sections against blood pool.

Nonetheless, the potential applications for such a device are broad, as many etiologies of cardiovascular disease exhibit regional heterogeneity. As intimated by Stendahl and colleagues, one potential application is localized inflammatory cell infiltration after acute myocardial infarction, where the severity and persistence of adverse inflammation can contribute to worse prognosis and remodeling.^5^,^11^ Clear delineation of regions with excessive inflammatory response may help to identify patients and regions that would most benefit from targeted therapy. Definitive characterization of the cellular substrate, as noted earlier, could also guide patients toward the most effective treatment based on the individual inflammatory pattern.

Ultimately, the greatest strength of the non-invasive or minimally invasive approach is the capability to combine regional diagnostics with regional therapy. Regional inflammation could be directly treated by anti-inflammatory agents, gene therapy to promote endogenous repair, or immunomodulatory therapy to target adverse remodeling processes.^13^,^14^ Such approaches could overcome the hazards of systemic immunomodulation. Alternatively, prior studies have reported alignment of nuclear cardiac images to catheter-based measures of electrophysiology, where the site of inducibility of ventricular arrhythmia corresponded to lower retention of norepinephrine analogues.^15^,^16^ Integration of the β-detection capability with electrode measurements could provide further insights into this pathogenesis and provide a clearer map for ablation. The clinical relevance of the β-detection catheter is intimately linked to its potential theragnostic applications, which require dedicated investigation.

Miniaturization to explore pathogenesis remains the realm of science fiction, the capability to visualize distinct regional differences in tissue substrate within the injured myocardium on a miniatured scale is an initial step toward tissue-level quantification and substrate delineation. The potential combination of such tissue-level imaging techniques with targeted delivery of cell-, gene-, or drug-based therapy bears clear potential for future modification of image-guided therapy. This catheter-based fantastic voyage provides a template for accurate study of regional tracer distribution, but many steps remain to bring such methodology to clinical practice.
